# Emerging fatal gout disease in Chinese goslings linked to acute kidney injury induced by novel goose astrovirus infection

**DOI:** 10.3389/fcimb.2024.1470808

**Published:** 2024-09-18

**Authors:** Zhuangli Bi, Xuan Lv, Zicheng Zhang, Linying Cai, Miao Zhang, Wanxiao Li, Yingying Ding, Huiwen Liu, Kang Yang, Yingqi Zhu, Guangqing Liu, Guijun Wang

**Affiliations:** ^1^ College of Animal Science and Technology, Anhui Agricultural University, Hefei, China; ^2^ Shanghai Veterinary Research Institute, Chinese Academy of Agricultural Sciences, Shanghai, China

**Keywords:** goose astrovirus, acute kidney injury, renal fibrosis, renal cell apoptosis, excessive inflammatory response

## Abstract

A novel goose astrovirus (GAstV) has broken out across China in recent years, causing widespread damage to the poultry industry. In goslings infected with GAstV, the leading cause of death is visceral gout. However, our understanding of the mechanism of gout formation in GAstV infection is largely inadequate. The aim of this study was to examine the pathogenicity of a GAstV strain and explore the molecular mechanisms of visceral gout caused by viral infection in goslings. The virulent GAstV strain HR2105/1 was effectively isolated from the visceral tissue of goslings in gout-affected areas. The whole genome of the HR2105/1 strain was sequenced and analyzed. Subsequently, we established a gosling gout models with experimental GAstV infection. Finally, we conducted a study on the mechanism of GAstV induced acute kidney injury. Phylogenetic analysis of the complete genome sequence showed that it was closely related to the strain circulating in China since 2016, and it was grouped within the GAstV-1 cluster. The clinical signs were reproduced by experimental infection of healthy goslings with the isolated strain and were found to be similar to those reported in clinical cases. Moreover, the virus exhibits strong renal tropism. Infection with the GAstV strain HR2105/1 was found to cause acute kidney injury, as evidenced by increased levels of uric acid and creatinine as well as severe pathological damage. Mechanistic experiments with Masson and Picrosirius Red staining revealed fibrosis in renal tissues after GAstV infection. Furthermore, TUNEL staining revealed that GAstV infection triggered renal cell apoptosis. Additionally, RT-qPCR revealed that GAstV infection caused an excessive inflammatory response by upregulating the expression of IL-1β, IL-6, IL-10, TGF-β, and iNOS in renal tissues. Overall, our findings demonstrate that GAstV infection causes renal damage by inducing renal cell apoptosis, fibrosis, and excessive inflammatory response, which subsequently leads to hyperuricemia and lethal visceral gout formation. This is the first systematic study on the etiology of lethal gout in goslings caused by GAstV infection, and we believe that the findings can guide vaccine development and therapeutic targets for GAstV-associated renal diseases.

## Introduction

A novel goose astrovirus (GAstV), which has caused substantial economic losses in the major goose-producing regions of China, is become a matter of growing concern (Niu et al., 2018; Zhang X. et al., 2018). First identified in Shandong and Anhui provinces in 2016, the novel GAstV spread quickly to other provinces in China and is associated with a mortality rate of approximately 20–50% (He et al., 2022; Q. Zhang et al., 2018). Infection with GAstV is mainly characterized by white feces, enlargements of leg joints as a result of urate deposits, and paralysis. Necroscopic examination reveals severe urate deposition in the leg joints and internal organs, including the ureters, kidney, gallbladder, and the surfaces of the heart and liver, eventually leading to lethal visceral gout (Tang, 2018). In fact, visceral gout is the leading cause of death in goslings infected with GAstV. However, the mechanism by which gout is induced by GAstV is unclear.

Gout development is a complex process involving multiple factors. Usually, gout is caused by high amounts of urate deposits in the form of sodium salts in tissues in and around the joints, abdomen, and organs, following long-standing hyperuricemia (Gibson, 2012). The mechanism of hyperuricemia mainly involves an imbalance in purine metabolism and urate excretion. Urate is mainly produced in the liver, and normal levels of blood urate are maintained through its excretion via the kidney and intestines. About two-thirds of the urate is excreted from the kidney, and the remaining one-third is excreted from the intestines (Mandal and Mount, 2015). It has been reported that reduced excretion of urate caused by renal dysfunction is the predominant cause of hyperuricemia in animals (Maiuolo et al., 2016). Moreover, some viruses, including infectious bronchitis virus and goose hemorrhagic polyomavirus, are known to cause gout, which is associated with infection-induced kidney injury (Ren et al., 2020; Tu et al., 2021). In this study, we have explored whether lethal visceral gout caused by GAstV infection is associated with kidney injury and its potential molecular mechanisms.

A number of potential pathophysiological pathways through which acute kidney injury (AKI) can develop under conditions of viral infection have been proposed based on experimental evidence. Of the pathways implicated, inflammation may be a key player in the pathogenesis of AKI caused by virus infection. For example, about 25% of patients hospitalized with COVID-19 have been reported to develop AKI, and the pathophysiology of AKI caused by SARS-COV-2 is thought to be associated with excessive local and systemic immune responses (Legrand et al., 2021; Sharma et al., 2020). Further, it has been reported that Zika virus infection can also induce AKI in both newborn and adult mouse models by activating the NLRP3 inflammasome and apoptosis, and this mechanism is considered to be responsible for renal disease caused by Zika infection (Liu et al., 2019). Apoptosis has been reported to be associated with AKI induced by virus infection in other studies too (da Mata et al., 2021). For instance, Chen et al. found that nephropathogenic infectious bronchitis virus (NIBV) infection can induce renal apoptosis in chickens by activating the GRP78/PERK/ATF-4 signaling pathway, thereby leading to kidney injury (Chen et al., 2022). Further, Middle East respiratory syndrome coronavirus infection, with concurrent acute respiratory distress syndrome, is typically associated with a high incidence of renal failure, in which viral infection-induced renal apoptosis plays a central role (Yeung et al., 2016). Additionally, virus infection-induced kidney fibrosis is another common pathological basis for AKI. In the case of GAstV infection, its ability to cause AKI and the related mechanisms that contribute to hyperuricemia and gout remain to be investigated.

Based on the gaps in the literature mentioned above, in the present study, we identified and isolated a lethal GAstV strain from diseased goslings in the field. Their whole viral genomes were sequenced and analyzed. Furthermore, 2-day-old goslings were infected with GAstV to establish a gout model using which we explored the possible mechanism of gout induced by GAstV infection and the related renal pathologies. We believe that our findings will provide new insights into the prevention and control of GAstV infection and the associated gout and renal pathways.

## Materials and methods

### Clinical and pathological investigations

In August 2021, an infectious disease was observed in a goose breeding farm in Hefei city, Anhui province, China. The mortality rates of the affected flocks were as high as 20%. Clinical autopsy revealed the deposition of urate in visceral organs and joint cavities, severe bleeding and enlargement of the kidneys, and urate deposition in the ureter. Tissue samples of the kidney and liver from four goslings were collected and shipped on ice to our lab to identify possible causative viral pathogens. The samples were stored at −80°C until use. RT-PCR and PCR assays were carried out to detect GAstV, the influenza virus, the Tembusu virus, and goose parvovirus using previously described primers and procedures (Li et al., 2021; Liao et al., 2023).

### Goose astrovirus isolation

To isolate GAstV from the samples, they were homogenized in sterile phosphate-buffered saline (PBS) and centrifuged at 8000 × g for 10 min. The supernatants were sterilized through a 0.22-μm filter (Millipore) and inoculated into the chorioallantoic membrane of 10-day-old goose embryos (0.2 ml per egg). The allantoic fluid and embryoid bodies of embryos that died after 24 h and those that survived until 5 days after inoculation were harvested under sterile conditions and stored at -80°C for further analysis.

### Immunofluorescence assay

The virus supernatants (200 μL) were inoculated into the cells of the chicken hepatocellular carcinoma cell line LMH and incubated for 1 h at 37°C in a 5% CO_2_ atmosphere to allow for adsorption. Following this, the medium was replaced with Dulbecco’s Modified Eagle Medium/Nutrient Mixture F-12 (DMEM/F12, New York, USA) containing 2% fetal bovine serum (Gibco, Carlsbad, CA, USA) and 1% penicillin-streptomycin. The cultures were incubated at 37°C in a 5% CO_2_ atmosphere. At 72 h after inoculation, LMH cells were fixed with a solution of cold acetone and methanol (1:1, v/v) at −20°C for 20 min. The cells were washed three times with PBS and blocked with 5% skimmed milk at 37°C for 1 h. They were then incubated with monoclonal antibody (diluted 1:200) against the GAstV VP27 protein, which was prepared in our lab (Zhang et al., 2022), at 37°C for 1 h. After three more rounds of washing with PBS, the cells were incubated with a fluorescent isothiocyanate-conjugated goat anti-mouse IgG (1:200 dilution; Solarbio, Beijing, China) at room temperature for 1 h in the dark. The cells were then stained with the nuclear stain 4′,6-diamidino-2-phenylindole (1:1000, Solarbio, Beijing, China) for 7 min at room temperature, washed three times with PBS, and examined using an inverted fluorescence microscope (Olympus IX53, Tokyo, Japan).

### Genome sequencing and genetic analysis

Viral RNA was extracted using a RNAeasy™ Animal RNA Isolation Kit (Beyotime, Shanghai, China), and cDNA was synthesized using the M-MLV Reverse Transcriptase (Promega, Madison, USA) according to the manufacturer’s instructions. The whole genome of GAstV was amplified using specific primers (**Table 1**). The amplified PCR products were subjected to agarose gel electrophoresis and excised from the agarose gel for purification, which was performed with the TIANgel Midi Purification Kit (Tiangen, Beijing, China), and for sequencing (Songon, Shanghai, China) to obtain accurate viral genome information. Sequence alignment and comparison were carried out using the DNAMAN software (Lynnon Corporation, CA), and phylogenetic analysis and p-distance calculation were performed with MEGA 7.0 using the neighbor-joining method (Kumar et al., 2016). Bootstrap values were calculated on 1,000 replicates of the aligned sequences.

**Table 1:** Primers used in this study.

Primer	Sequence 5′- 3′
GAstV-F1-F	AAACAGCGATATGGCGGC
GAstV-F1-R	TGTACGACTTAGTCCAAGGAACG
GAstV-F2-F	CACAGAGGCTACAATGCT
GAstV-F2-R	CCTTCAACAACGACAATGG
GAstV-F3-F	CAGTCCCTGTACAGATTTTA
GAstV-F3-R	TCAACTTGTTCATCCTTTAC
GAstV-F4-F	AGATTGATGAAGCCATTGAG
GAstV-F4-R	CAGCCCGCCGTTCTGTCTGT
GAstV-F5-F	AGGCTGTATCAGATATTGAT
GAstV-F5-R	TCATTTTGTCATTAACGGG
GAstV-F6-F	TCTGGTGAGTGGCGGACCGA
GAstV-F6-R	TGTCATAACAGCCCACCAATTGTGT
GAstV-F7-F	ACAACTGGACAAGGTACC
GAstV-F7-R	TTTGCGGATTTTAAATGC
IL-1β-F	GAGGCCGAGGAGCAGGGACTTT
IL-1β-R	AGGACTGTGAGCGGGTGTAGCG
IL-6-F	AGATGGTGATAAATCCTGATG
IL-6-R	CGGTTTTCTCCATAAATGAAGT
IL-10-F	TGCCTCCACTTGTCTGACC
IL-10-R	TCCTCCATGTAGAACCGCATC
TGF-β-F	TCTCGGAGCAGCGGATAG
TGF-β-R	AGCACGGGCAATGTAAGC
iNOS-F	GAACAGCCAGCTCATCCGATA
iNOS-R	CCCAAGCTCAATGCACAACTT
β-actin-F	GCTATGTCGCCCTGGATTTC
β-actin-R	CACAGGACTCCATACCCAAGAA

### Pathogenicity assessment of GAstV in goslings

To determine the pathogenicity of GAstV, 80 one-day-old goslings were randomly divided into two groups of 40 each and housed in separate specific pathogen-free isolators. The goslings in the experimental groups were inoculated with 1 mL of the isolated GAstV strain, designated as HR2105/1 by subcutaneous injection in the neck. The control group was inoculated with equal doses of PBS at the same injection site. Water and food were autoclaved before feeding and automatically refilled. Clinical signs and gross and microscopic lesions were recorded after infection. Five goslings in each group were necropsied at 1, 3, 6, 9, 12, and 15 days post-inoculation (dpi). In addition, serum samples were collected from all the selected goslings and stored at − 20°C. These samples were later used to detect and record changes in biochemical parameters, including uric acid (UA) and creatinine (CRE). Tissue samples of the heart, liver, kidney, brain, and spleen were collected and stored at − 80 °C and used for virus detection, histopathological analysis (hematoxylin-eosin [HE] staining), immunofluorescence assay (IFA), and immunohistochemistry (IHC staining). Any goslings nearing death on the same day were collected for additional research material. After 15 days of virus infection, the remaining geese were euthanized by intravenous injection of sodium pentobarbital (100 mg/kg body weight).

The gosling infection experiments described in this study were approved by the Animal Care and Use Committee of Anhui Agricultural University, China, and conducted using humane procedures.

### Quantitative real-time PCR

The levels of GAstV in the tissue samples of the heart, liver, kidney, brain, and spleen were measured by quantitative real-time PCR. Viral RNA was extracted using a QIAamp Viral RNA Mini Kit (Qiagen, Hilden, Germany), and cDNA was synthesized using the HiScript Q RT SuperMix for qPCR (+gDNA wiper) (Vazyme, Nanjing, China) according to the manufacturer’s instructions. Real-time PCR was conducted on an ABI PRISM 7500 sequence detection system and ChamQ Universal SYBR qPCR Master Mix (Vazyme, Nanjing, China). The reaction mixtures were denatured at 95°C for 3 min, and this was followed by 40 two-step cycles of 95°C for 10 s and 60°C for 30 s. The PCR primers used were as follows: forward: 5’-AAGCAACAGACAGAACG-3’, reverse: 5’-TATCCGCCAGAAGAGAG-3’. A pMD-19T vector (TaKaRa, Beijing, China) containing an inserted ORF1b gene of GAstV was used to construct standard plasmids. A standard curve was generated using serially diluted plasmid standards at concentrations of 10^0^–10^7^ copies/μL. The copy number of the GAstV RNA was calculated using the standard curve method.

The concentrations of IL-1β, IL-6, IL-10, TGF-β, iNOS, and β-actin in the supernatants of the kidney tissues were determined by qPCR using specific primers (**Table 1**). qPCR analyses of mRNA were performed on the ABI PRISM 7500 sequence detection system in a ChamQ Universal SYBR qPCR Master Mix kit (Vazyme, Nanjing, China). The housekeeping gene β-actin was used as an internal control for gene expression normalization. Relative transcript levels were analyzed using the 2^−ΔΔ^CT method (Livak and Schmittgen, 2001).

### Histopathological examination and immunohistochemistry

The kidney tissue samples were fixed in 10% neutral buffered formalin for 48 h at room temperature, dehydrated through an ethanol series, clarified in xylene, and embedded in paraffin. The samples were then sliced serially into 4-μm sections, stained with hematoxylin and eosin (HE), and examined under a light microscope. Masson staining and Picrosirius Red staining were performed according to the protocols provided with the kits (Beijing Solarbio Science & Technology Co. Ltd., Beijing, China).

Immunohistochemistry was performed using anti-VP27 protein monoclonal antibodies produced in our lab. Tissue sections were prepared as previously described in this section, and the prepared sections were deparaffinized, hydrated, heated in a water bath for antigen retrieval, and blocked with 3% hydrogen peroxide. Subsequently, immunohistochemical staining was performed as described previously (Madapong et al., 2020).

### TUNEL assay

The TUNEL procedure was applied to the kidney sections to detect DNA fragmentation as an index of apoptosis. Apoptosis in the kidney tissue section was evaluated as previously described in the One Step TUNEL Apoptosis Assay Kit (Beyotime Biotech., Shanghai, China). TUNEL-positive cells were observed under a fluorescence microscope at ×200 magnification (HB050; Zeiss, Hamburg, Germany).

### Serum UA and CRE level detection

The serum levels of UA and CRE were measured using commercially available kits (Nanjing Jiancheng Bioengineering Institute, Nanjing, China) according to the manufacturer’s protocols.

### Statistical analysis

Data are presented as mean ± standard deviation. The statistical software SPSS 19.0 (IBM Corp., Armonk, NY, USA) was used to perform all the statistical analyses. Differences between the control group and experimental group were analyzed by the Student t-test. p ≤ 0.05 was considered to indicate statistically significant differences.

## Results

### Goose astrovirus isolation

**Figure 1:**
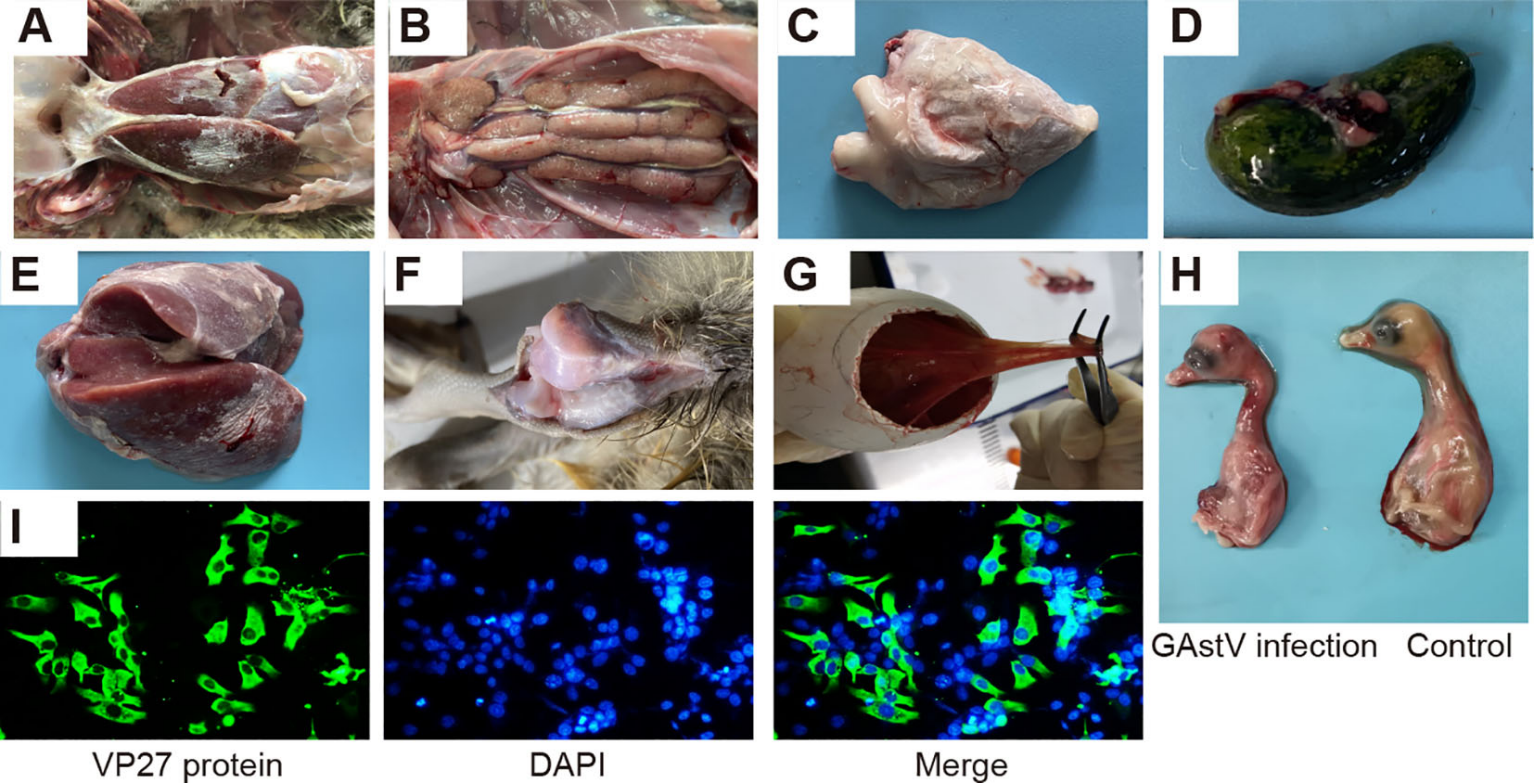
Isolation of a novel goose astrovirus strain: HR2105/1. **(A)** Gross lesions of morbid goslings from commercial goose farms. **(B)** Renal enlargement and urate deposition. **(C)** Urate deposition on the surface of the heart. **(D)** Urate deposition in bile sacs. **(E)** Urate deposition in the liver. **(F)** Urate deposition in the articular cavity. **(G, H)** Infected goose embryos at 48–72 h post-infection, showing allantoic membrane thickening and subcutaneous hemorrhage. **(I)** Indirect immunofluorescence assay demonstrating infection of LMH cells with GAstV isolated from infected geese.

In 2021, a suspected novel goose astrovirus infection occurred in a goose farm in Anhui province, China. The affected goslings were found to display noticeable pathological alterations, including typical visceral gout or articular gout with the deposition of urate crystals on the surfaces of the liver and heart (**Figures 1A, C, E**) and swelling and pale appearance of the kidneys with white urate deposition in the ureters (**Figure 1B**). In addition, distended bile sacs with abundant urate particles (**Figure 1D**) and white urate deposits in the articular cavity were also observed (**Figure 1F**). Samples were collected, and virus isolation was performed using the conventional procedure. As shown in **Figures 1G** and **H**, after three serial passages, the goose embryos died at 48–96 h after infection due to diffuse hemorrhage accompanied by edematous thickening of the chorioallantoic membrane.

Allantoic fluid containing the virus was obtained from the embryos and used to infect LMH cells. Immunofluorescence analysis was performed using a monoclonal antibody specific to the VP27 protein of GAstV. This analysis revealed the presence of abundant green fluorescence signals localized in the cytoplasm of the LMH cells (**Figure 1I**). This isolated GAstV strain was designated as the HR2105/1 strain.

### Genome sequencing and sequence analysis

**Figure 2:**
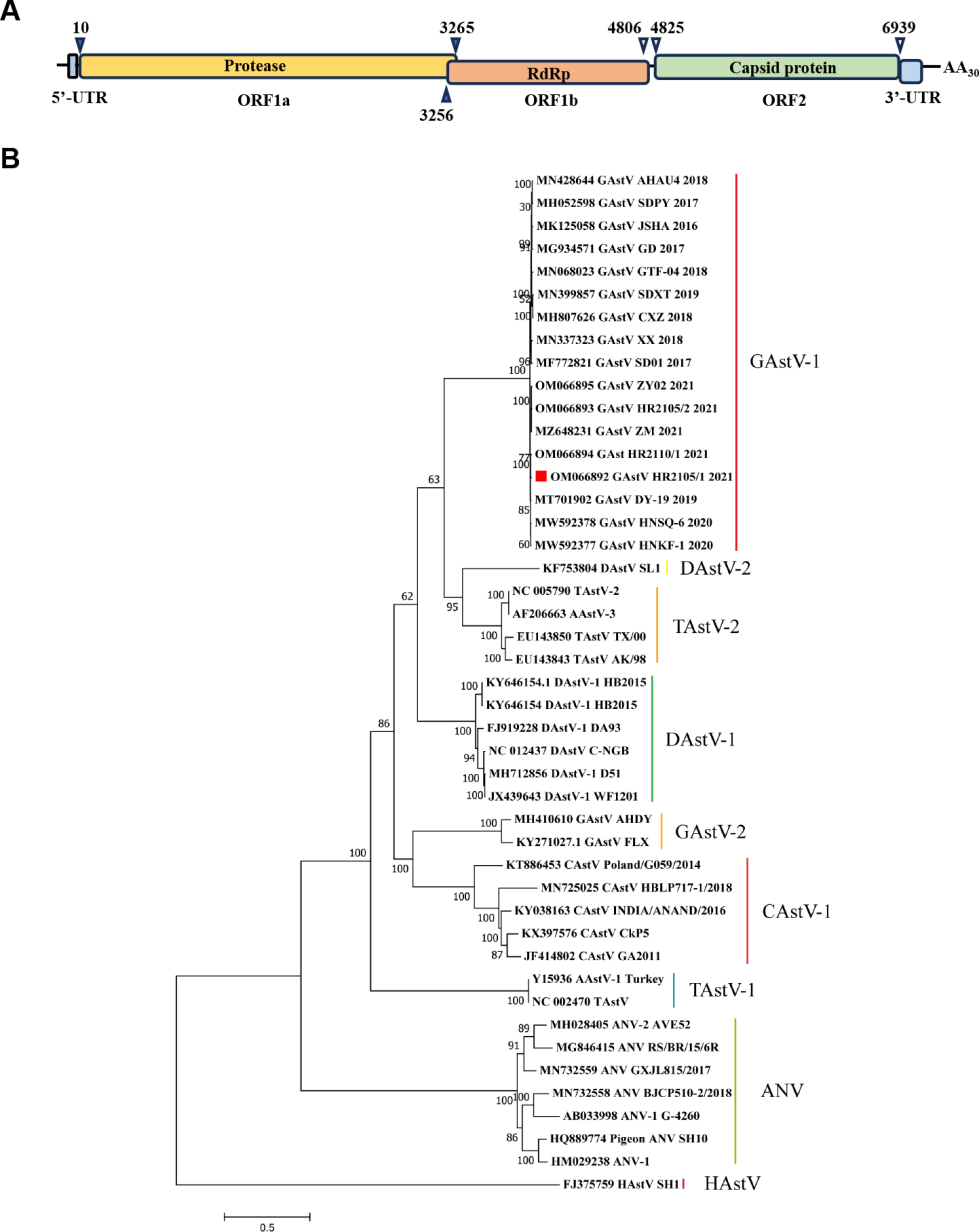
Genomic and phylogenetic analysis of the isolated goose astrovirus. **(A)** Predicted genome organization of GAstV HR2105/1. The nucleotide position of the three predicated ORFs (including stop codons) in the genome is shown. **(B)** Phylogenetic tree based on the whole genome of Astrovirus.

To further investigate the molecular characteristics of the GAstV strain HR2105/1, the complete genome was amplified with the primers listed in **Table 1**. On assembling the sequences, the genome of the HR2105/1 strain was found to be 7175 nucleotides long, flanked by 10 nucleotides of the 5′ NTR sequence and 236 nucleotides of the 3′ NTR sequence. The genome of the HR2105/1 strain contains three open reading frames: ORF1a, ORF1b, and ORF2. ORF1a, which is 3255 nt long, and ORF1b, which is 1551 nt long, encode the nonstructural proteins of GAstV, while ORF2, which is 2115 nt long, encodes the viral capsid protein (**Figure 2A**). The genome sequences of GAstV obtained in the present study were deposited in GenBank under accession number OM066892.

To determine the phylogenetic relationships of the newly identified GAstV-HR2105/1 strain with other GAstVs and avian astroviruses (AAstVs), the available complete genome data of all GAstVs and representative AAstVs deposited in GenBank were downloaded and used for genetic and phylogenetic analyses. The results showed that the GAstV strain HR2105/1 was highly divergent from all other AAstVs but showed a relatively close relationship to novel GAstV strains, with high bootstrap support (100%) (**Figure 2B**). These results suggest that the HR2105/1 strain we isolated is a novel astrovirus that causes fatal gout in goslings.

### Establishing gosling gout models with experimental GAstV infection

**Figure 3:**
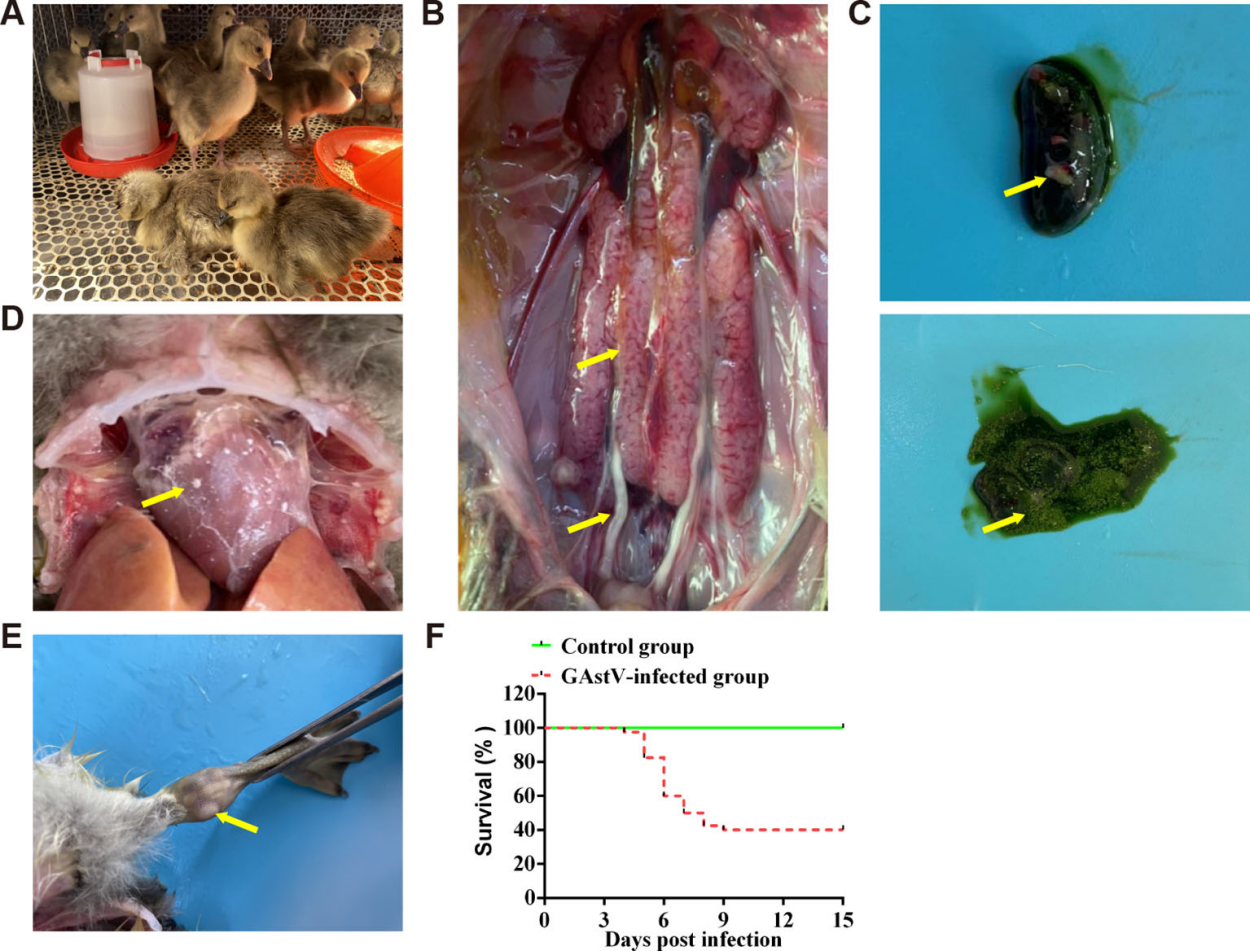
Gross lesions of dead goslings after inoculation with GAstV HR2105/1. **(A)** The infected goslings appear depressed and lethargic. **(B)** Urate deposition, swelling, and bleeding in the kidney. **(C)** Urate deposition in bile sacs. **(D)** Heart showing obvious urate deposition. **(E)** Obvious urate particles in the joint lumen. **(F)** Survival curve of infected goslings.

To further evaluate the virulence of the isolate in goslings, an infection experiment was performed using one-day-old goslings. Infected goslings exhibited signs of depression from 3 dpi, and this sign persisted for 3–4 days (**Figure 3A**). Necroscopic analysis showed that goslings in the infected group exhibited features that were typical of visceral gout or articular gout, including the deposition of urate crystals in the ureters and bile sacs, on the surface of the heart, and in the articular cavity (**Figures 3B–E**). Goslings in the infection group began to die at 4 dpi, and the mortality rate of the group at 15 dpi was 60% (**Figure 3F**). These results indicate that the isolated strain was virulent and could successfully induce gout in goslings.

### Strong renal tropism of GAstV

**Figure 4:**
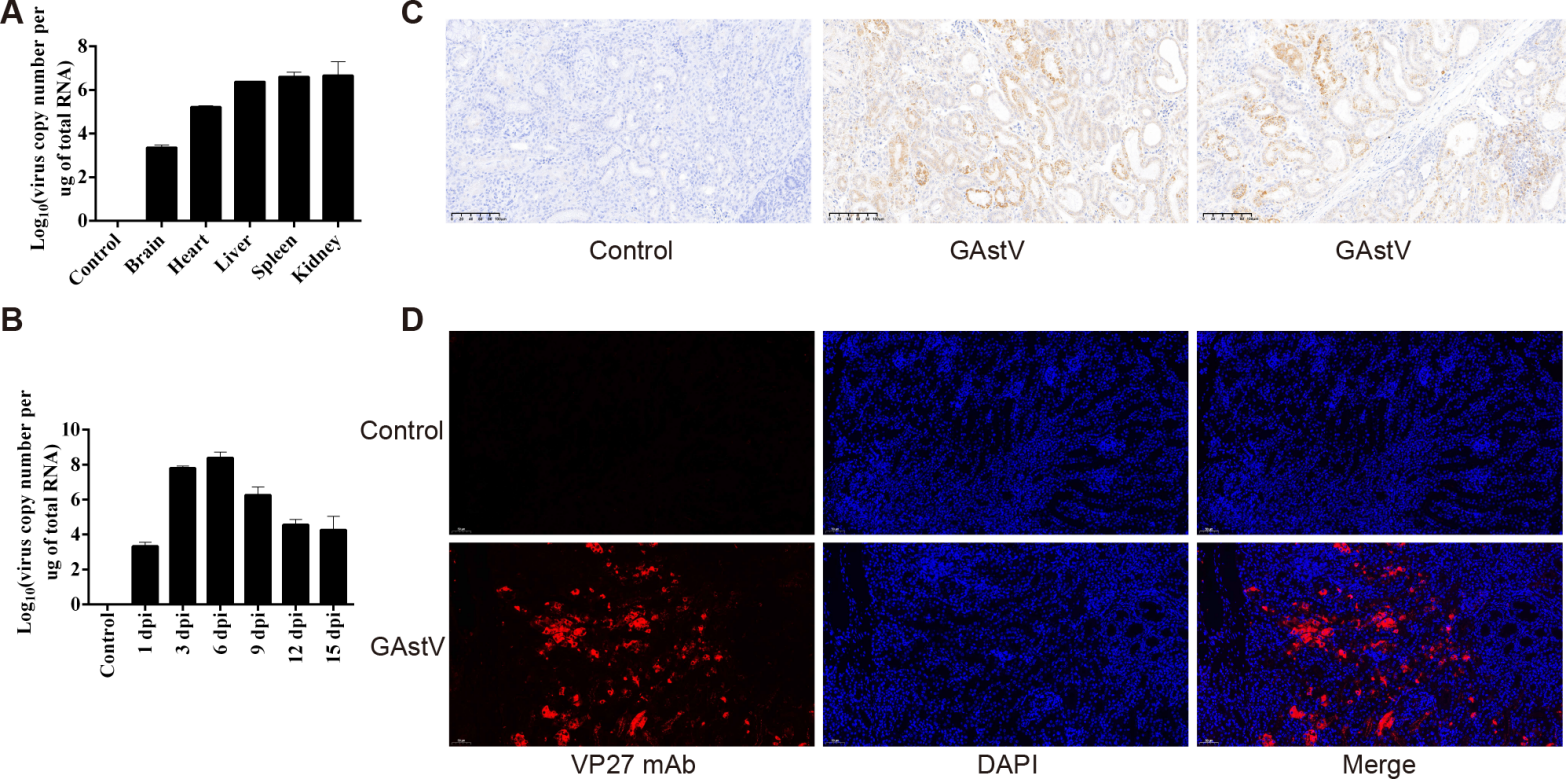
Strong tropism of GAstV in the kidney of goslings. **(A)** Detection of viral load in different organs of dead goslings by RT-qPCR. **(B)** Detection of viral load in the kidney tissues of infected goslings by qRT-PCR at different times. **(C)** Immunohistochemical staining of GAstV-infected kidney tissues using a monoclonal antibody against the GAstV VP27 protein prepared in our lab. **(D)** Indirect immunofluorescence assay of GAstV-infected kidney tissues using a monoclonal antibody against the GAstV VP27 protein prepared in our lab.

We detected the virus copy number in different organs, including the heart, liver, kidney, brain, and spleen, to determine whether GAstV exhibits renal tropism. As shown in **Figure 4A**, no GAstV was detected in the control group samples, whereas the virus was found in all the examined tissues of the infected goslings. Among these tissues, the kidney is one of the tissues with the highest virus titer. This implies that the kidney is one of the main target organs of GAstV. Furthermore, RT-qPCR assays were used to detect viral load in kidney tissues at regular intervals after the viral challenge (at 1, 3, 6, 9, 12, and 15 dpi). As shown in **Figure 4B**, on day 6 after the challenge, the virus level in the kidney reached its peak and then began to decline slowly until the end of the experiment. The GAstV antigen in the kidneys of infected goslings was detected by IHC using an anti-VP27 monoclonal antibody. GAstV was observed in both the renal glomerulus and tubule (**Figure 4C**). Further, the presence of GAstV in the kidneys was confirmed by immunofluorescence analysis (**Figure 4D**). Taken together, these data indicate that the kidney was the main target organ of GAstV, and gout induced by GAstV may be associated with strong tropism of GAstV in the kidney.

### GAstV infection-induced AKI in goslings

**Figure 5:**
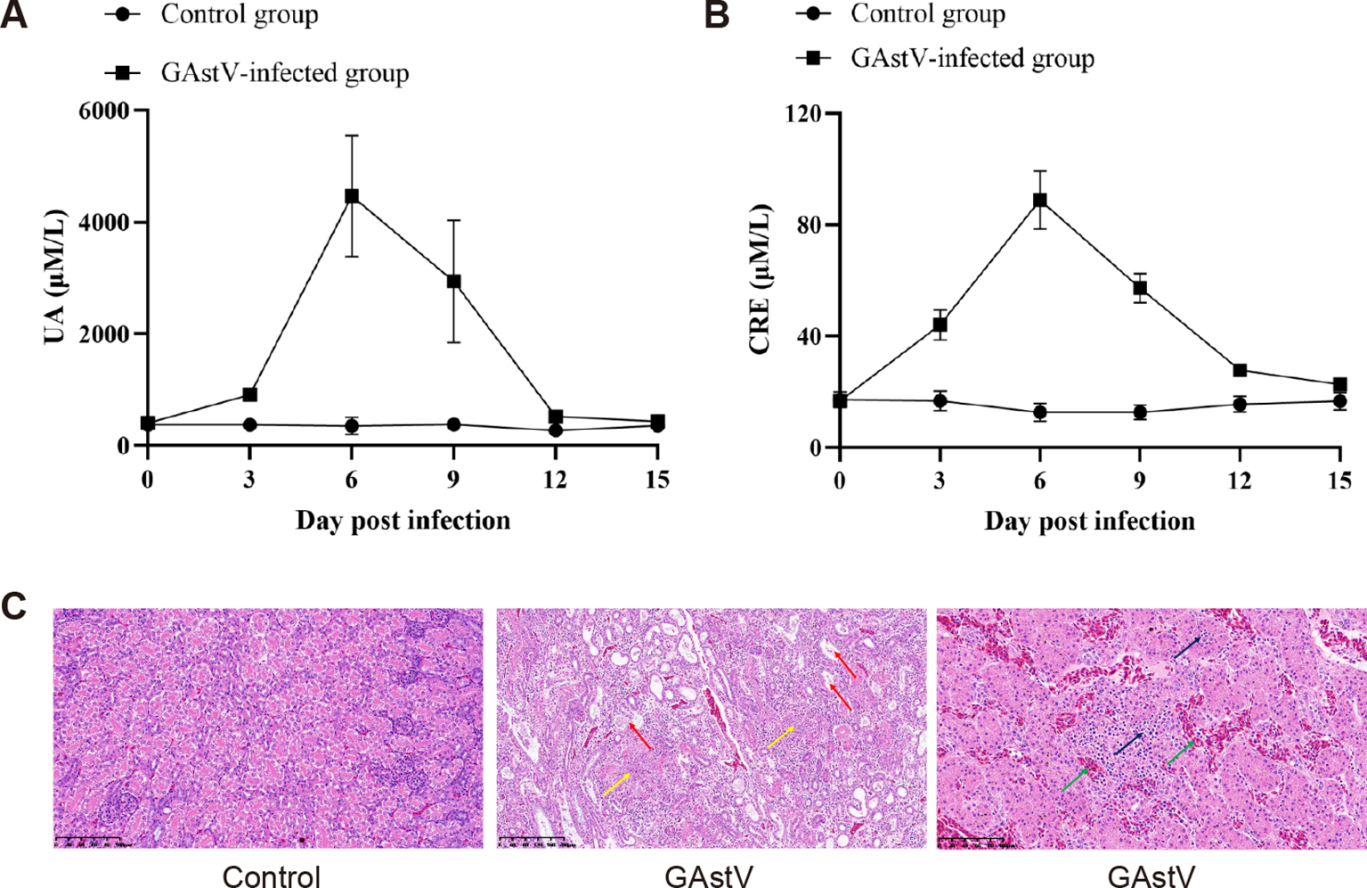
GAstV infection-induced acute kidney injury. **(A)** The serum content of uric acid (UA) was tested in GAstV-infected goslings using an UA assay kit at 0, 3, 6, 9, 12, and 15 days post-infection. **(B)** The serum creatinine (Scr) concentration was tested in GAstV-infected goslings using a creatinine assay kit at 0, 3, 6, 9, 12, and 15 days post-infection. **(C)** HE staining of kidney tissue from infected and control goslings. Histopathological changes in the kidney at 6 dpi after experimental infection of goslings with GAstV. Obvious urate crystals (yellow arrows) can be seen in multiple parts of the kidneys of infected goslings, accompanied by degeneration, necrosis, and shedding of renal tubular epithelial cells (red arrows), renal interstitial bleeding (green arrows), and lymphocyte infiltration (black arrows).

As the kidney was the main target organ of GAstV, we further investigated whether GAstV infection affected renal function. First, as the most important indices of renal function in animals, the UA and CRE concentrations were detected in gosling serum samples obtained at different time points. As shown in **Figures 5A** and **B**, the UA and CRE concentrations in the kidney reached their peak on day 6 after the challenge and then began to slowly decline. In addition, HE staining clearly showed degeneration and necrosis of renal epithelial cells and inflammatory cell infiltration (**Figure 5C**). All these data suggest that GAstV infection can result in AKI, although the mechanisms via which GAstV induces AKI remain largely unknown and still need to be investigated.

### GAstV infection-induced kidney fibrosis

**Figure 6:**
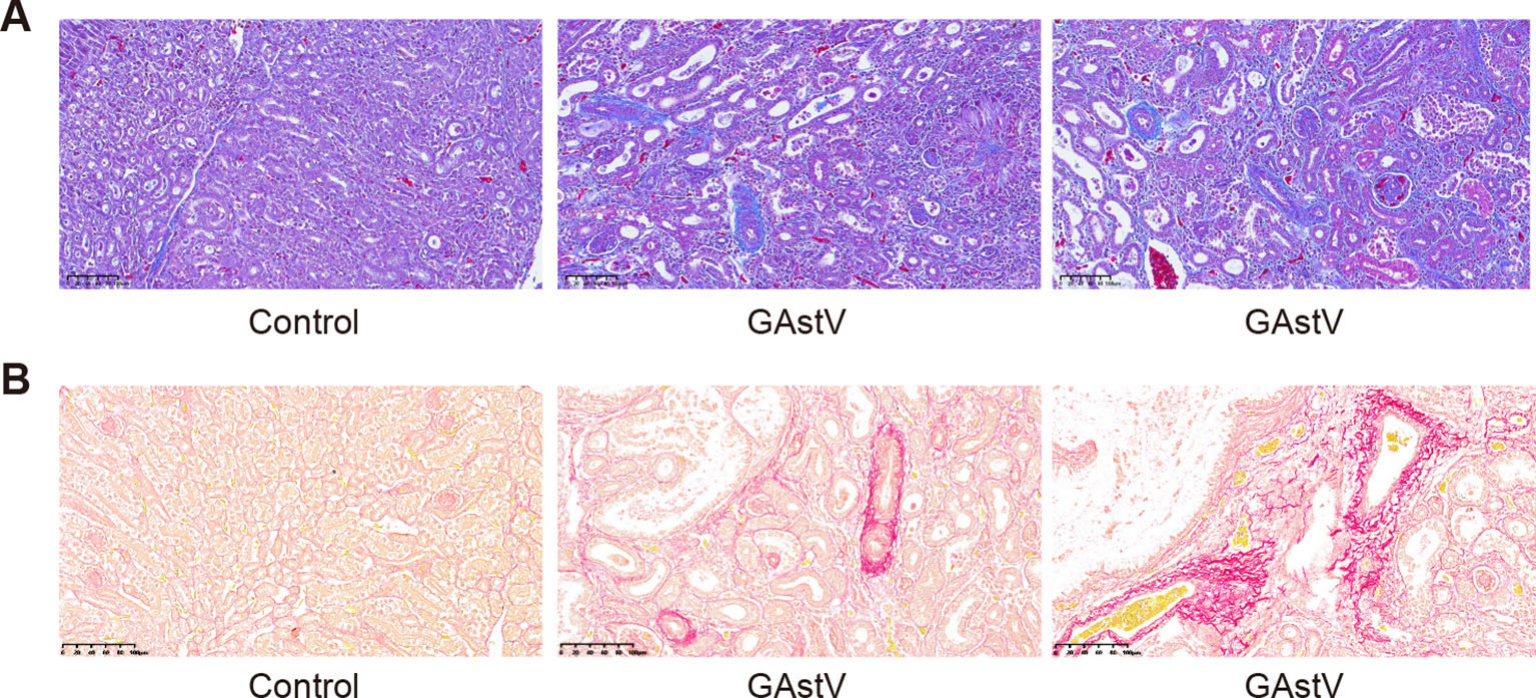
GAstV infection-induced renal fibrosis. **(A)** Masson staining images showed tubulointerstitial fibrosis (blue staining) in the kidneys of GAstV-infected goslings at 3 6 days post-infection. **(B)** Picrosirius Red staining displaying different levels of proliferation of kidney fibrous tissue in GAstV-infected goslings at 6 days post-infection.

Kidney fibrosis induced by virus infection is a common histological manifestation of a functional decline in the kidney (Soomro et al., 2016). Therefore, we also tried to detect whether GAstV infection causes renal fibrosis through Masson and Picrosirius Red staining. The results showed that there were no significant changes in either stain in the control groups, while increased tubulointerstitial fibrosis and collagen fibers were observed in the infected group tissues (**Figures 6A, B**). These results imply that infection with the goose astrovirus GAstV can cause renal fibrosis.

### GAstV infection-induced cell apoptosis

**Figure 7:**
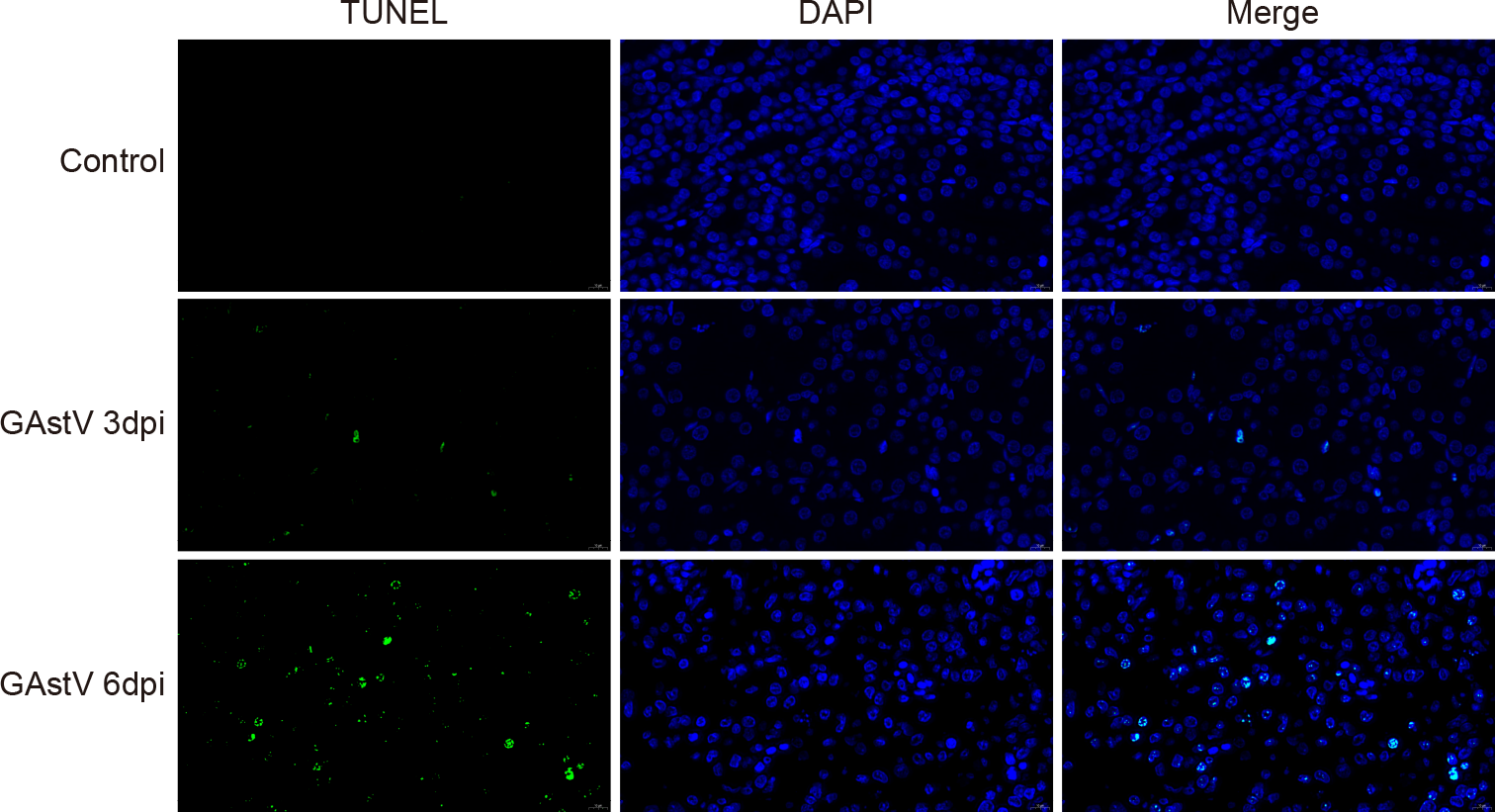
GAstV infection-induced renal cell apoptosis. Representative TUNEL-stained histological sections of kidneys from GAstV-infected gosling at 3 and 6 days post-infection (100×).

Apoptosis induced by virus infection is one of the main causes of AKI (Turpin et al., 2022). To verify whether GAstV infection caused apoptosis in the kidney, fixed kidney tissues were selected for apoptosis detection. No apoptotic signals were detected in the control group with TUNEL staining, while obvious apoptotic signals (green fluorescence) were visible in the GAstV infection group. Further, the apoptotic signal at 6 dpi was higher than that at 3 dpi (**Figure 7**). Thus, our results show that GAstV infection can cause apoptosis in renal tubular epithelial cells.

### GAstV infection-induced excessive inflammatory response in the kidney

**Figure 8:**
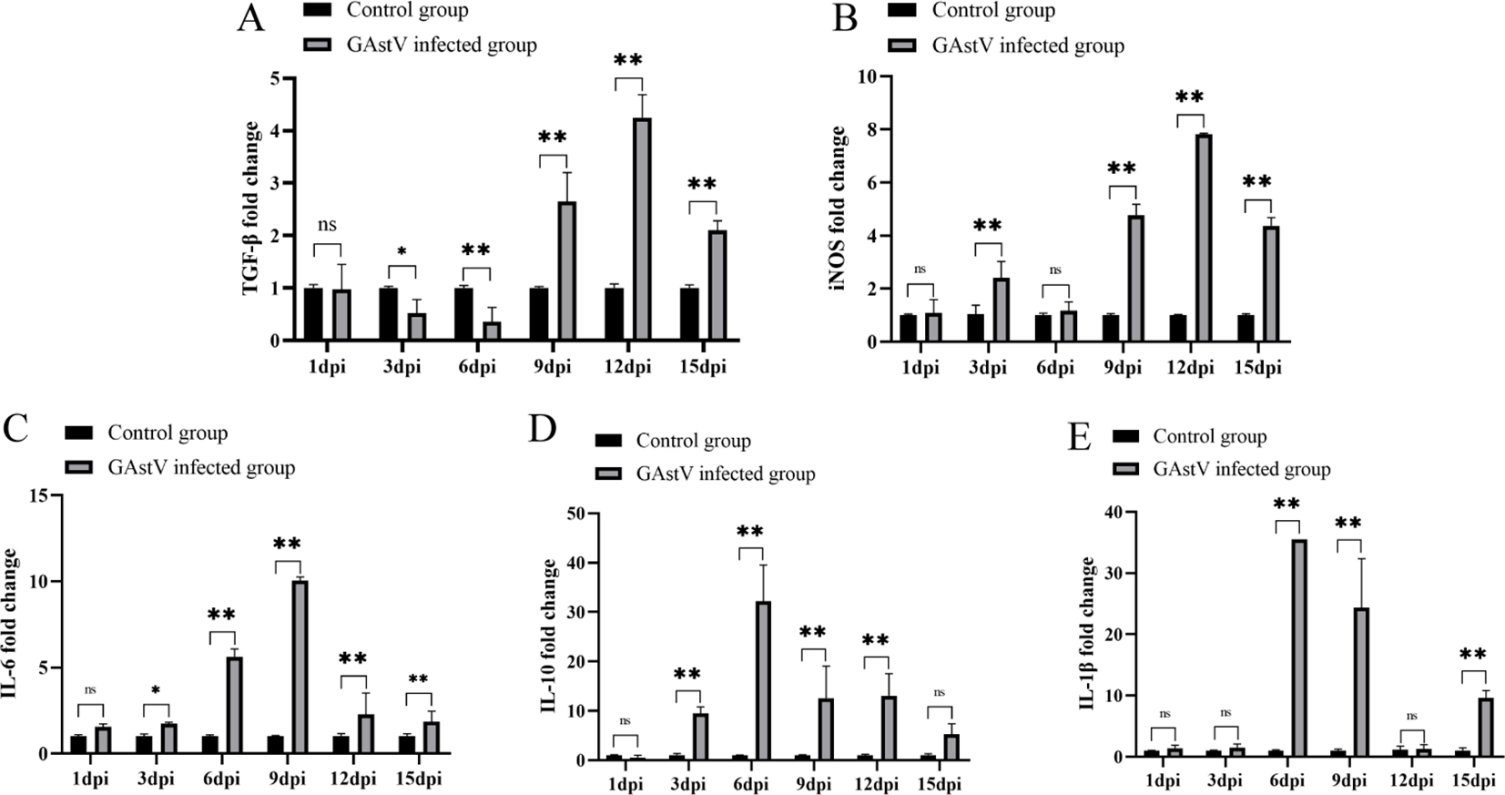
GAstV infection-induced excessive inflammatory response in the kidney. The relative mRNA expression levels of TGF-β **(A)**, iNOS **(B)**, IL-6 **(C)**, IL-10 **(D)**, and IL-1β **(E)** in kidney tissues of goslings were measured by RT-qPCR at 1, 3, 6, 9, 12, and 15 days post-infection. Data were presented as mean ± SD (n = 6), *p < 0.05, **p < 0.01 vs. Control group. ns indicates no significant difference.

Excessive inflammation is not only a feature of AKI but also an essential mechanism that promotes kidney deterioration, which triggers hyperactivation of immune cells in the kidney and results in the release of large amounts of inflammatory mediators, such as cytokines and chemokines, further exacerbating kidney injury (Sun et al., 2023). To further evaluate the influence of GAstV infection on the inflammatory response, the levels of TGF-β, iNOS, IL-6, IL-10, and IL-1β in the kidney tissues of infected goslings were measured by qRT-PCR. As shown in **Figures 8A–E**, the expression levels of the pro-inflammatory factors iNOS, IL-6, IL-10, and IL-1β were significantly increased in the kidney tissues in the infected group compared with the control group. Interestingly, compared with the control group, the expression level of TGF-β was significantly downregulated before 6 dpi and significantly upregulated after 6 dpi. The above results indicate that GAstV infection caused an excessive inflammatory response in the kidneys of goslings.

## Discussion

In the present study, we isolated a novel goose astrovirus strain, HR2105/1, from the tissue samples of infected goslings that exhibited typical gout sign. Phylogenetic analysis conducted in this study revealed that the GAstV strain HR2105/1 was highly divergent from all other known AAstVs, but showed a relatively close relationship to novel GAstV strains. Further, immunohistochemical examination revealed that HR2105/1 infection can cause AKI. Importantly, renal fibrosis, renal epithelial cell apoptosis, and an excessive inflammatory response were identified as mechanisms of infection with the GAstV strain HR2105/1. We believe that our findings will be useful for investigating the pathogenesis of GAstV infection in goslings with gout and for developing effective preventive measures and control strategies.

GAstV was first detected in 2016 and has since rapidly spread to Shandong, Jiangsu, Anhui, Guangxi, Guangdong, and other provinces in China, causing significant economic losses to the poultry industry (De Benedictis et al., 2011; Ejaz et al., 2005; Jin et al., 2018; Liu et al., 2018; Niu et al., 2018; Q. Zhang et al., 2018). Severe visceral gout and articular gout are typical characteristics of GAstV infection and the leading cause of high mortality (20%–50%) in infected goslings (Niu et al., 2018). However, little information is available about the relationship between GAstV infection and goose gout, or the mechanisms underlying this infection. Previous studies have demonstrated that virus infection may be involved in the outbreaks of gout in poultry. For example, NIBV infection was found to result in enlarged and pale kidneys and monosodium urate crystal deposition in the renal tubules and ureters in chickens (Cook et al., 2012; Gonzales-Viera et al., 2021). Further, chickens inoculated with waterfowl influenza viruses displayed swollen kidneys, as evident from the accentuated lobular patterns observed, urate deposits in the pericardial sac, and microscopic lesions in the kidneys that were consistent with visceral gout (Slemons et al., 1990). In our study, goslings infected with the GAstV strain HR2105/1 were found to have a mortality rate of 60% and exhibited typical signs of visceral gout, that is, the deposition of urate crystals in the ureters and bile sacs, on the surfaces of the liver and heart, and in the articular cavity. This is fulfilling Koch’s Postulates, proving that the GAstV is indeed the pathogen causing gout in goslings. Moreover, we found that this virus has strong renal tropism in goslings and could even cause AKI. Based on these findings, we deduced that the renal damage caused by GAstV infection resulted in a reduction in uric acid excretion and an increase in serum uric acid levels, which led to hyperuricemia, consequently contributing to gout. However, the mechanism underlying the kidney damage induced by the virus is unclear and needs to be investigated.

Inflammatory response is a “double-edged sword.” A moderate inflammatory response is conducive to virus elimination, but an excessive inflammatory response can induce a “cytokine storm” and contribute to viral pathogenesis (Carty et al., 2021). Increasing clinical evidence shows that excessive inflammation may be a key player in the pathogenesis of AKI caused by virus infection. For example, it was found that SARS-COV-2 infection triggers the activation of multiple inflammatory pathways and results in a cytokine storm involving IL-6, C-reactive protein, TGF-β signaling, and complement activation to cause AKI (Chen et al., 2021). Further, Li et al. found that NIBV promoted cytokine release through the TLR7/NF-κB signaling axis, thus causing kidney injury (Li et al., 2022). In line with the previous finding, in this study, too, excessive elevation of pro-inflammatory factors, including IL-1β, IL-6, IL-10, TGF-β, and iNOS, was induced by the GAstV strain HR2105/1 in kidney tissues. These findings, combined with the histopathological changes observed, imply that excessive inflammatory response plays an important role in virus-induced kidney damage.

Apoptosis, as one of the terminal pathways of cell death, is a typical form of programed cell death that participates in antiviral responses aimed at limiting the spread of a virus (Carruthers et al., 2007; Chen et al., 2022). However, renal apoptosis is also considered to be a prominent pathological feature in most forms of renal injury (Liang et al., 2017). For example, a Zika virus infection was found to induce AKI by activating apoptosis through suppressing Bcl-2 expression (Liu et al., 2019). Further, it has been proposed that interventions targeting renal apoptosis may be an effective strategy for the treatment of renal injury (Yang et al., 2018). In our study, TUNEL staining was used to evaluate apoptosis in renal cells, and the number of TUNEL-positive cells was found to be significantly higher in the infected group samples than in the control group samples, with renal tubular epithelial cells accounting for the majority of the stained cells. The presence of GAstV in renal tubular epithelial cells implies that the virus may be directly responsible for the apoptosis of renal tubular epithelial cells. Collectively, these results indicate that renal cell apoptosis caused by GAstV infection may be an important cause of AKI.

Renal fibrosis is the final common and devastating pathway for kidney diseases and is characterized by glomerulosclerosis and tubulointerstitial fibrosis (Zhou et al., 2012). Injured renal tubular epithelial cells are the primary drivers of renal fibrosis, and the maladaptive repair of injured cells after AKI leads to dysfunction in kidney structure and function (Ferenbach and Bonventre, 2015). Recently, some evidence has indicated that virus infection can induce kidney tubulointerstitial fibrosis, which could be attributed to tissue damage induced by the inflammatory response (Liu et al., 2019). In this study, Picrosirius Red staining indicated the presence of fibroplasia, and Masson’s staining clearly demonstrated tubulointerstitial fibrosis in the kidney. These observations are suggestive of damage to the kidney parenchyma after GAstV infection. This could have resulted from the upregulation of pro-inflammatory factors, among which TGF-β is known as a strong profibrotic cytokine (Su et al., 2020).

**Figure 9:**
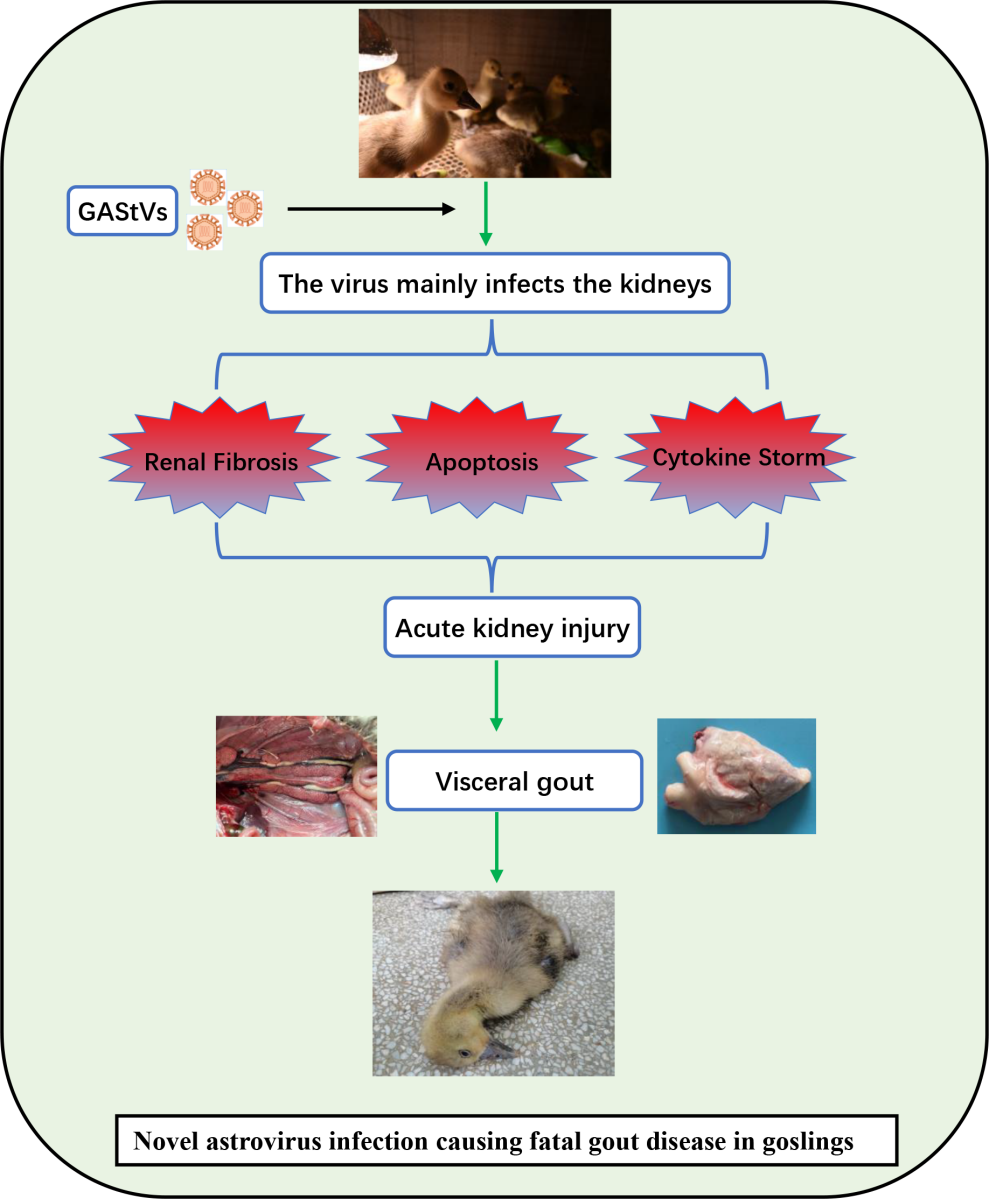
Schematic diagram depicting the virulence mechanisms of the GAstV strain HR2105/1. The strain has strong renal tropism and can cause AKI in the goslings by inducing kidney fibrosis, renal epithelial cell apoptosis, and an excessive inflammatory response. With regard to the disease pathway, kidney injury leads to the obstruction of uric acid excretion, which leads to hyperuricemia and, ultimately, fatal visceral gout.

In conclusion, we successfully isolated a novel goose astrovirus strain and used it to induce typical gout in experimental goslings. GAstV has the ability to replicate in various organs and also exhibits strong renal tropism. Furthermore, we demonstrated that GAstV infection can cause AKI, thereby reducing uric acid excretion and resulting in hyperuricemia. More specifically, we found that GAstV infection-induced AKI may be caused by renal tubular epithelial cell apoptosis, excessive inflammation in renal tissue, and renal fibrosis (**Figure 9**). These findings have increased our understanding of the mechanism by which GAstV causes lethal gout in goslings and provide a reference for future research into such infections.

## Data Availability

The datasets presented in this study can be found in online repositories. The names of the repository/repositories and accession number(s) can be found in the article/supplementary material.
